# Therapeutic effects of human gingiva-derived mesenchymal stromal cells on murine contact hypersensitivity via prostaglandin E_2_–EP_3_ signaling

**DOI:** 10.1186/s13287-016-0361-9

**Published:** 2016-08-02

**Authors:** Pei Li, Yuming Zhao, Lihong Ge

**Affiliations:** Department of Pediatric Dentistry, Peking University School and Hospital of Stomatology, 22 Zhongguancun Avenue South, Haidian District, Beijing, 100081 People’s Republic of China

**Keywords:** Gingiva-derived mesenchymal stromal cells, Immunomodulation, Contact hypersensitivity, Local injection, Therapeutic administration, Prostaglandin E_2_–EP_3_ pathway

## Abstract

**Background:**

The immunomodulatory and anti-inflammatory functions of human gingiva-derived mesenchymal stromal cells (GMSCs) have been demonstrated in contact hypersensitivity (CHS) models; however, their therapeutic effect during the late phase of CHS has been poor.

**Methods:**

The murine CHS model was induced by applying oxazolone to the ears of mice. Mesenchymal stromal cells were applied via two methods (intravenous or local injection) at three time points: 1 day before sensitization, 1 day before challenge, or 1 h after challenge. Prostaglandin E_2_ (PGE_2_) and sulprostone were administered subcutaneously 1 h after challenge.

**Results:**

The application of GMSCs, bone marrow mesenchymal stem cells, and adipose-derived stem cells all effectively suppressed CHS; however, GMSC treatment exhibited the greatest efficacy. Local injection of GMSCs led to a more marked attenuation of CHS compared with intravenous injection, especially during the late phase of CHS, and this manifested as decreased infiltration of inflammatory cells, suppression of the levels of various proinflammatory cytokines, reconstruction of the disrupted Th1/Th2 balance, and upregulation of regulatory T cells in the allergen contact areas. Pretreatment with indomethacin significantly abrogated the GMSC-mediated immunosuppressive effects, while PGE_2_ application reversed the effects of indomethacin pretreatment of GMSCs. Moreover, GMSC administration promoted the expression of EP_3_, a prostaglandin E receptor, and the application of sulprostone, an agonist of EP_3_, significantly attenuated CHS to a similar degree as that of GMSC administration.

**Conclusions:**

GMSCs have reproducible and powerful immunomodulatory functions. Local injection of GMSCs is the superior mode for therapeutic application. PGE_2_–EP_3_ signaling plays an important role in the immunomodulatory functions of GMSCs in murine CHS.

**Electronic supplementary material:**

The online version of this article (doi:10.1186/s13287-016-0361-9) contains supplementary material, which is available to authorized users.

## Background

Mesenchymal stem cells (MSCs), or mesenchymal stroma cells, are adult progenitor cells present primarily in bone marrow and probably in most adult tissues. MSCs, including human bone marrow-derived MSCs (BMSCs) and adipose-derived stem cells (ASCs), have been shown to possess potent immunomodulatory and anti-inflammatory functions by inhibiting the proliferation and activation of multiple types of innate and adaptive immune cells or promoting the differentiation of regulatory T cells (Tregs) [1–4]. This manifests as decreased production of proinflammatory cytokines and upregulation of growth factors and soluble factors with anti-inflammatory functions [5, 6]. Human gingiva-derived MSCs (GMSCs) were first isolated and identified by Zhang et al*.* in 2009 [7] and are considered a new source of MSCs with a promising future in regenerative medicine [8, 9]. Recent studies reported that human GMSCs have immunomodulatory properties similar to those of BMSCs, including inhibition of T-cell proliferation and activation, enhancement of Treg generation, and polarization of M2 macrophages [7, 8, 10]. Specifically, GMSCs can be isolated and obtained readily, maintain a normal karyotype and telomerase activity over long-term culture, display a stable phenotype, and proliferate rapidly in vitro [11, 12]. These characteristics render GMSCs a potential novel immunotherapeutic agent. Recently, BMSCs [5, 13, 14] and ASCs [13–15] have been used for the treatment of a variety of immune-related and inflammation-related diseases. However, the different effects between treatments using GMSCs and other types of MSCs have not yet been explored, which might limit their application. This study therefore first compared the immunomodulatory capabilities of BMSCs, ASCs, and GMSCs.

Murine contact hypersensitivity (CHS) is widely used as a model for allergic contact dermatitis (ACD). One of the most common diseases caused by repeated skin exposure to contact allergens, ACD is classified as a type IV or a delayed type hypersensitivity reaction. The CHS model comprises two phases: the sensitization phase, in which skin dendritic cells take up antigens, migrate to regional draining lymph nodes, and stimulate the activation and differentiation of allergen-specific T cells; and the elicitation phase, in which effector T cells evoke immune inflammation upon exposure to antigens [16]. The first-line treatment for ACD is topical application of corticosteroids [17], which only partially alleviate the local symptoms. There is thus an urgent need for a more effective therapeutic tool. Su et al*.* [17] demonstrated that intravenous injection of GMSCs attenuates the appearance of CHS in mice before antigen sensitization and challenge. This suggests that GMSCs administered prophylactically could home to, and function at, the site of local inflammation in tissue. However, GMSC administration after challenge was less effective for CHS attenuation compared with before antigen sensitization and challenge. Thus, evidence is lacking for the efficacy of therapeutic administration of GMSCs. This study therefore focused on the therapeutic administration of GMSCs, particularly on how to increase the efficacy of therapeutic administration.

Although convincing findings for the therapeutic effects of MSCs on a variety of immune-related and inflammation-related diseases have been reported, how to deliver MSCs to targeted sites of inflammation in a timely fashion and in sufficient numbers to optimize their therapeutic effect has attracted increasing levels of attention. Rather than intravenous MSC administration, local MSC administration may be preferable. Multiple studies have demonstrated that topical or subcutaneous application of MSCs to cutaneous wounds promotes their repair in both mice [18–20] and humans [18, 21]. Substantial research has also focused on treatment with locally applied MSCs for complications of diabetes, including polyneuropathy (MSC intramuscular injection) [22], ischemic hind limb (MSC intramuscular injection) [23], foot ulcerations (MSC subcutaneous injection) [24], and diabetic wounds (MSC subcutaneous injection) [25]. Against this background, to explore the therapeutic effects of novel strategies of MSC application in mice with CHS, we compared local and intravenous GMSC administration in our study.

Prostaglandin E_2_ (PGE_2_) is metabolized from arachidonic acid by sequential catalysis of COX [16]. PGE_2_ functions in allergic inflammation by interacting with PGE receptors which are a family of four subtypes of G protein-coupled proteins (EP_1_, EP_2_, EP_3_, and EP_4_). For example, PGE_2_ promotes an inflammatory response through Th1 and Th17 cell expansion via EP_4_ or EP_1_ signaling [26, 27]; however, PGE_2_ suppresses skin allergic inflammation via EP_3_ [16, 28], which is expressed abundantly in the skin [29]. To clarify the role of EP signaling in allergic skin inflammation, this study focused on the interaction between GMSCs and the PGE_2_–EP pathway in a CHS model.

Nonsteroidal anti-inflammatory drugs (NSAIDs) that block COX would be expected to suppress allergic inflammation in the skin. However, NSAIDs usually have no significant effect on inflammation in CHS in experimental animals or clinical patients, which is attributed to increased levels of leukotriene B4 [16].

Our research aims to optimize MSC therapy in mice with CHS. First, to identify the preferable MSC type we compared the immunomodulatory functions of isolated GMSCs with the most commonly used MSCs (BMSCs and ASCs) in a CHS model. Second, to optimize the administration methods of MSCs, we explored novel strategies of MSC application, specifically local injection. Last, we investigated the possible mechanisms of the immunomodulatory functions of MSCs, especially the relationship to the PGE_2_–EP pathway.

## Methods

### Cell culture

GMSCs [7], ASCs [30], and BMSCs [31] were isolated following established protocols (Additional file 1) approved by the Institutional Review Board of Peking University School and Hospital of Stomatology (PKUSSIRB-201311108). Clinically healthy gingiva was collected from routine dental procedures as a remnant or discarded tissue. Alveolar bone marrow was aspirated from patients undergoing routine dental implant placement at Peking University School and Hospital of Stomatology. Human adipose tissues were obtained from healthy patients who underwent liposuction surgery for aesthetic reasons at Peking University Third Hospital, following approved guidelines set by the Health Science Center, Peking University. Human tissue samples were obtained after informed consent from all of the donors.

Cells obtained from the second to sixth passages were used in the experiments. GMSCs were pretreated with 5 M indomethacin (Sigma, St. Louis, MO, USA) in vitro for 24 h and then used for the animal experiments.

### GMSC characterization

MSCs were defined by differentiation into osteogenic, adipogenic, and chondrogenic phenotypes [7] and expression of the MSC markers CD73, CD105, CD146, STRO1, and CD34, as determined by flow cytometry.

### Animal treatments

Animal experiments in this study were approved by the laboratory animal welfare ethics branch of the Institutional Review Board of Peking University (LA2015052). BALB/c mice (male, 8–10 weeks old) were obtained from Vital River Laboratories (Beijing, China) and housed in groups in the animal facility of Peking University (Beijing, China). All animal care and experiments were performed using institutional protocols approved by the Institutional Review Board of Peking University School and Hospital of Stomatology.

Our mouse CHS model is similar to that described in previous studies [17]. Initially, 25 μl of 2 % oxazolone in acetone:olive oil (4:1) was applied to the right ear. The ears were then challenged with 15 μl of 2 % oxazolone 7 days after the first sensitization. An equivalent amount of vehicle (acetone:olive oil (4:1)) was administered to the left ear as a control. Ear thickness was measured 5 mm away from the ear margin before and 24 h after the challenge in a blinded fashion, and the difference was used as a parameter of ear swelling.

Initially, five groups of mice (*n* = 10 per group) were used to compare the treatment effect of GMSCs, BMSCs, ASCs, and corticosteroid. Different types of MSCs (2 × 10^6^ cells per mouse) were applied by intravenous injection (injected into the tail vein) 1 day before sensitization. Skin-derived fibroblasts were injected by the same method as the CHS control. Approximately 30 mg of 0.025 % triamcinolone acetonide acetate cream (Qiangsheng, Beijing, China), a commonly used corticosteroid, was topically applied 2 h after challenge and then at 12-h intervals as a treatment control for each ear.

Next, GMSCs (2 × 10^6^ cells per mouse) were applied by two methods, intravenous or local injection (see Additional file 2), at three time points: 1 day before sensitization, 1 day before initiation or challenge, and 1 h after challenge (*n* = 10 mice for each method at each time point). For local injection, we chose three injection points on the bottom skin of the ears. The distance from the injection site to the ear margins was >3 mm. Skin-derived fibroblasts (2 × 10^6^) were injected by the same method as the CHS control.

We also applied 16,16-dimethyl-PGE_2_ at four different dosages (5, 10, 15, and 20 μg/kg) subcutaneously into CHS mice 1 h after challenge (*n* = 5 mice for each dosage). GMSCs were pretreated with 5 μM indomethacin in vitro to block PGE_2_ release completely and referred to as indomethacin-pretreated GMSCs (IGMSCs). Then 2 × 10^6^ skin-derived fibroblasts, GMSCs, IGMSCs, IGMSCs + PGE_2_, and sulprostone (0.1 mg/kg; Cayman Chemical, Ann Arbor, MI, USA) were subcutaneously applied to five groups of mice (*n* = 5 mice per group) 1 h after challenge.

Mice were sacrificed on day 2 after challenge, and ear samples were harvested for further analysis.

### Histomorphological analysis

Hematoxylin and eosin staining was performed on paraffin wax-embedded sections for histological examination. Immunohistochemical staining of collagen type II was performed using an antibody specific to human FceRIa. Immunohistochemical staining was performed using antibodies specific for mouse CD11b and human leukocyte antigen (HLA) (Abcam, Cambridge, UK) in paraffin sections of ears. Isotype-matched control antibodies (eBiosciences, San Diego, CA, USA) were used as negative controls.

### Enzyme-linked immunosorbent assay and western blot analysis

Cytokine concentrations in serum were assessed using enzyme-linked immunosorbent assay (ELISA) kits (eBioscience), and data were normalized by protein concentration. Western blot analysis of ear tissue was performed using antibodies specific to mouse tumor necrosis factor alpha (TNF-α), interleukin-6 (IL-6), IL-4, interferon-gamma (IFN-γ), IL-10, nuclear factor-kappa B (NF-kB), p65, Foxp3, CD4, transforming growth factor beta (TGF-β), inducible nitric oxide synthase (iNOS), EP_1_, EP_2_, EP_3_, EP_4_, and β-actin (Abcam).

### Statistical analysis

Data were collected from at least three independent experiments and analyzed using SPSS software (version 16.0; SPSS, Inc., Chicago, IL, USA) and GraphPad Prism version 5 (GraphPad Software Inc., La Jolla, CA, USA). After testing the normality of the data and confirming the applicability of these tests, one-way analysis of variance (ANOVA) was used to compare the means of multiple groups and Student’s *t* test was used to compare the means between two groups (e.g., intravenous vs local injection). Differences between groups were considered statistically significant at *p* < 0.05. Power analysis with NCSS-PASS (version 11.0; NCSS, Kaysville, UT, USA) was performed to ascertain whether the sample size was sufficient within the allowable error rate (α = 0.05, 1 – β = 0.9) after obtaining the data. The sample size of our research provides over 85 % power to detect differences among the means versus the alternative of equal means using an *F* test with a 0.05 significance level, which verifies the significance of our results.

## Results

### GMSC characterization

**Fig. 1:**
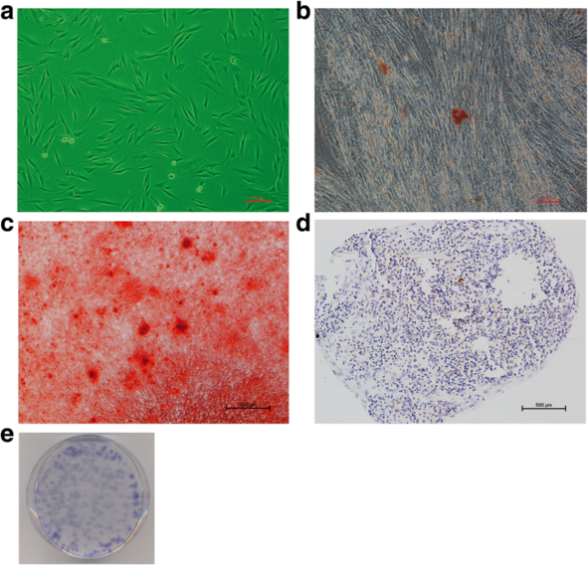
Characterization of human GMSCs. GMSCs showed a spindle-shaped, fibroblast-like morphology **a**; multilineage differentiation potency including adipogenesis **b**, as identified by Oil Red O staining; osteogenesis **c**, as identified by Alizarin Red S staining; chondrogenesis **d**, as identified by immunohistochemical staining of collagen type II; and colony-forming ability **e**, as identified by Toluidine Blue staining

**Fig. 2:**
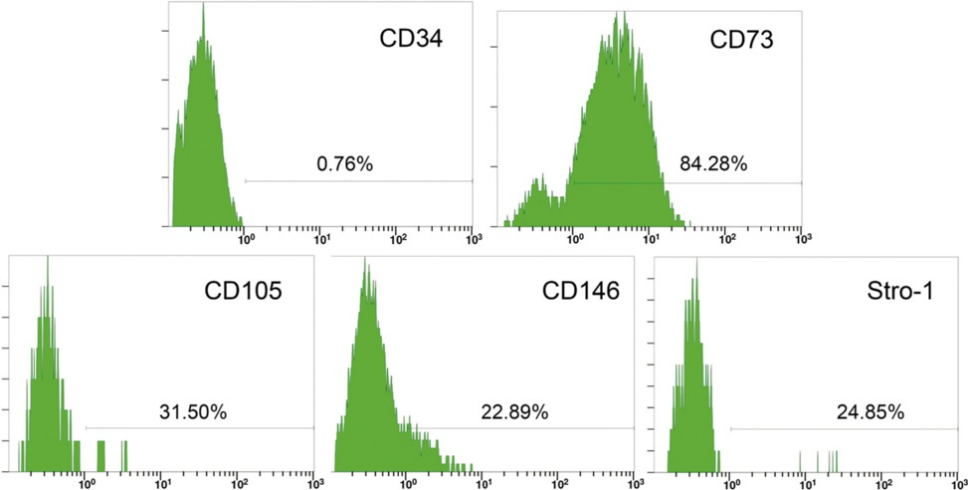
Flow cytometry of GMSCs. GMSCs expressed low levels of CD34 (0.76 %), and expressed CD73 (84.28 %), CD105 (31.50 %), CD146 (22.89 %), and STRO1 (24.85 %). *n* = 3 independent samples/donors

The population of nonepithelial progenitor cells isolated from normal gingival tissues showed a spindle-shaped, fibroblast-like morphology (Fig. 1a), colony-forming abilities (Fig. 1e), adherence to plastic, and multilineage differentiation potency, including adipogenesis (Fig. 1b), osteogenesis (Fig. 1c), and chondrogenesis, which is characterized by specific collagen II staining (Fig. 1d). The cells demonstrated low expression of CD34 (0.76 %) and expression of CD73 (84.28 %), CD105 (31.50 %), and the stem cell markers CD146 (22.89 %) and STRO1 (24.85 %), by flow cytometry (Fig. 2). Although the expression of CD73 and CD105 did not fit the minimal criterion for human MSCs—that is, more than 95 % of the MSC population must express CD105 and CD73, as proposed by the Mesenchymal and Tissue Stem Cell Committee of the International Society for Cellular Therapy [32]—the expression of other surface antigens including CD34, CD146, and STRO1 did fit this criterion. Moreover, these progenitor cells formed adherent clonogenic cell clusters confirmed by the CFU-F, and had multipotent differentiation potential. The cells used in our research thus had the basic characteristics of MSCs. According to the nomenclature in related published research [7, 8, 10, 17], we named the population of nonepithelial progenitor cells gingiva-derived mesenchymal stromal cells (GMSCs).

### Therapeutic effects of various MSC administration methods

**Fig. 3:**
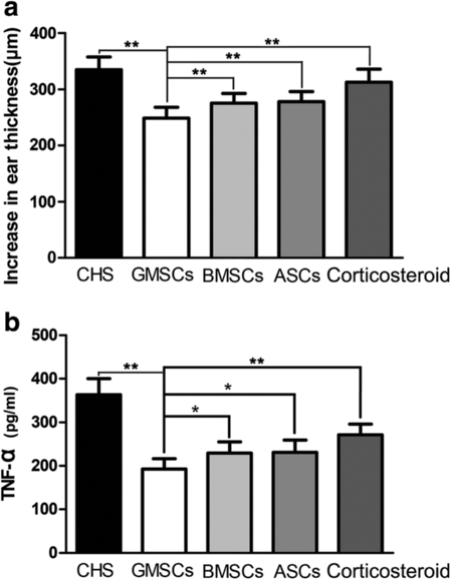
Therapeutic effects of the administration of various types of MSCs. **a** GMSC treatment led to significant reduction of ear thickness, compared with corticosteroid, ASC, and BMSC treatments. *n* = 10 independent animals/group. **b** ELISA showed that GMSC treatment led to a significant decrease in TNF-α levels compared with corticosteroid, ASC, and BMSC treatments. *n* = 5 independent animals/group. *p < 0.05; **p < 0.01. *CHS* contact hypersensitivity, *GMSC* gingiva-derived mesenchymal stromal cell, *BMSC* bone marrow-derived mesenchymal stromal cell, *ASC* adipose-derived mesenchymal stroma cell

First, we compared the immunomodulatory functions of GMSCs, BMSCs, and ASCs in the CHS model to identify the preferable type of therapeutic MSC. In our experiment, 24 h after challenge, GMSC, ASC, and BMSC treatments all led to statistically significant attenuation of CHS compared with the concentration and dosage of topical corticosteroid we used. GMSC treatment exhibited the greatest efficacy, followed by ASC and BMSC treatments (*p* < 0.01) (Fig. 3a). Consistent with these results, ELISA demonstrated that GMSC, ASC, and BMSC treatments resulted in significant decreases in the expression of TNF-α compared with corticosteroid treatment, and GMSC treatment led to a further significant decrease in TNF-α expression compared with ASC and BMSC treatments (*p* < 0.05) (Fig. 3b). GMSCs were therefore focused on in the following experiments because of their greater efficacy against CHS.

**Fig. 4:**
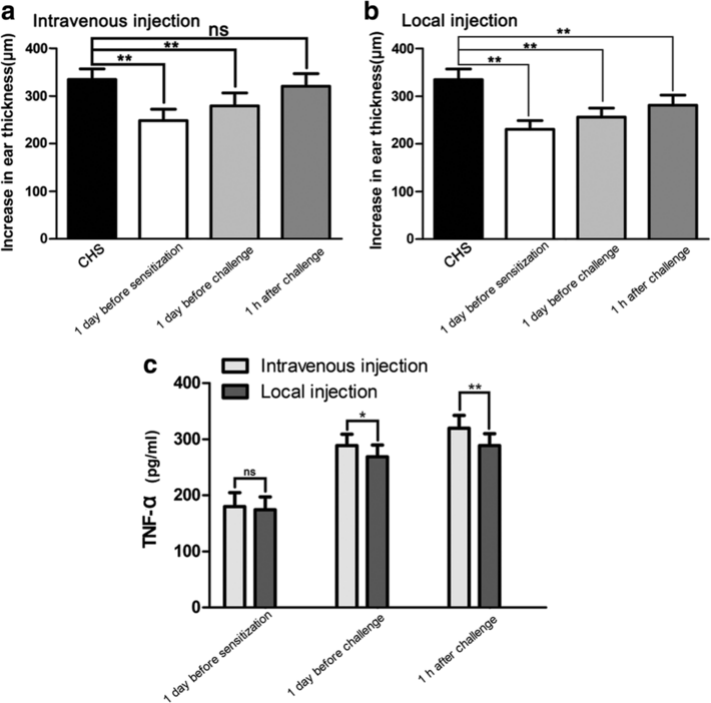
Therapeutic effects of various MSC administration methods. **a** Intravenous injection 1 h after challenge resulted in no significant reduction of ear thickness, but intravenous injection 1 day before sensitization and 1 day before challenge resulted in significant reduction of ear thickness compared with CHS mice. *n* = 10 independent animals/group. **b** Local injection 1 day before sensitization, 1 day before challenge, and 1 h after challenge consistently led to significant reduction of ear thickness. *n* = 10 independent animals/group. **c** ELISA of TNF-α production showed that local GMSC injection 1 day before challenge and 1 h after challenge significantly reduced TNF-α levels compared with intravenous injection. *n* = 5 independent animals/group. *p < 0.05; **p < 0.01; *ns* nonsignificance. *CHS* contact hypersensitivity

We then explored the therapeutic effects of different methods of MSC application at different stages of disease. The efficacy of GMSC treatment at different stages of CHS showed that GMSC infusion 1 day before sensitization and 1 day before challenge consistently led to significant reductions in ear thickness compared with CHS mice, regardless of the injection method (*p* < 0.01) (Fig. 4a, b). Intravenous GMSC infusion 1 h after challenge showed no significant reduction in ear thickness compared with CHS mice (Fig. 4a). However, local GMSC infusion 1 h after challenge led to a significant reduction in ear thickness compared with CHS mice (*p* < 0.01) (Fig. 4b). Moreover local and intravenous GMSC injection 1 day before sensitization resulted in no significant difference in TNF-α expression, but local GMSC injection 1 day before challenge and 1 h after challenge led to significantly lower TNF-α production compared with intravenous injection (*p* < 0.05) (Fig. 4c). Additional file 3 shows this in more detail. In summary, intravenous injection 1 day before sensitization and 1 day before challenge had a marked effect on CHS; however, local injection showed greater efficacy regardless of the injection time. In subsequent experiments we used the time point of 1 h after challenge, corresponding to the aforementioned therapeutic administration of GMSCs, unless indicated otherwise.

### Therapeutic effects of intravenous and local injection of GMSCs 1 h after challenge

**Fig. 5:**
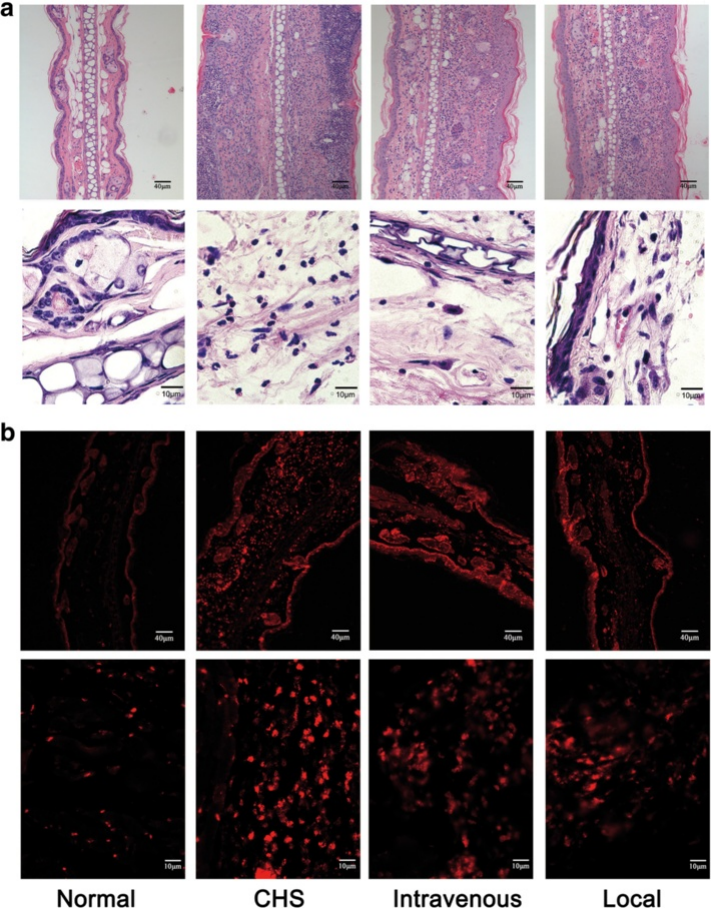
Histological images of ears following injection of GMSCs 1 h after challenge. **a** Hematoxylin and eosin-stained images of ears. **b** Immunofluorescence staining specific for mouse CD11b of ears. *n* = 3 independent animals/group. *CHS* contact hypersensitivity

**Fig. 6:**
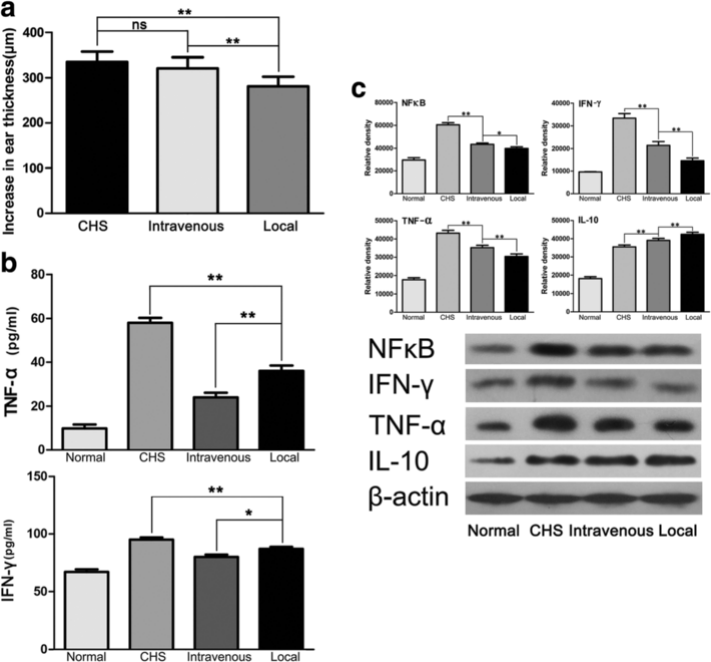
Comparison of the inflammatory degree of intravenous and local GMSC injection 1 h after challenge. **a** Measured ear thickness. *n* = 10 independent animals. **b** Level of TNF-α and IFN-γ in mouse serum detected by ELISA. *n* = 5 independent animals/group. **c** Western blot showed a significant increase in the level of IL-10 and significant decreases in the levels of NF-kB, TNF-α, IL-6, and IFN-γ in ear tissue lysates of intravenously and locally treated mice. *n* = 3 independent animals/group. *p < 0.05; **p < 0.01; *ns* non significance. *CHS* contact hypersensitivity

The time point of 1 h after challenge was focused on because it has a greater bearing on clinical practice. Mice were divided into the following groups: normal, CHS, intravenous injection, and local injection (*n* = 10 per group). Marked attenuation of CHS appearance was shown by hematoxylin and eosin staining (Fig. 5a). The number of CD11b-labeled inflammatory cells decreased markedly after both intravenous and local injection of GMSCs. Moreover, local injection resulted in a greater reduction of the number of CD11b-labeled inflammatory cells than did intravenous injection (Fig. 5b). The changes in ear thickness were consistent with the degree of inflammation (Fig. 6a). ELISA showed that intravenous and local GMSC injection resulted in significantly lower levels of TNF-α and IFN-γ in mouse serum compared with CHS mice, and intravenous GMSC infusion led to significantly lower levels of TNF-α and IFN-γ than did local infusion (Fig. 6b). Western blot showed a significant decrease in the expression of NF-kB p65 and proinflammatory cytokines, including TNF-α and IFN-γ, in tissue lysates of treated ears when compared with those of untreated CHS. However, local injection led to a greater decrease in the expression of these factors than did intravenous injection (Fig. 6c). At the same time, GMSC intravenous or local injection increased the expression of the anti-inflammatory cytokine IL-10 (Fig. 6c).

**Fig. 7:**
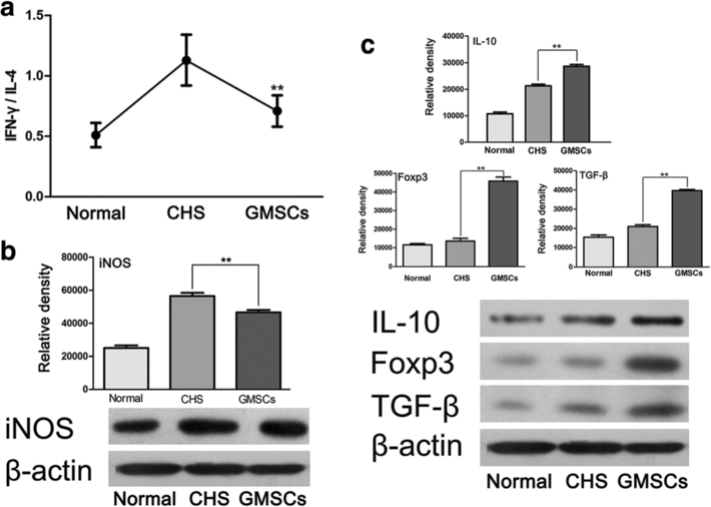
Changes in some markers related to inflammation after GMSC infusion. **a** IFN-γ/IL-4 ratio decreased significantly after GMSC injection. *n* = 5 independent animals/group. **b** Western blot showed a decrease in the expression of iNOS in tissue lysates of treated ears when compared with those of untreated CHS. *n* = 3 independent animals/group. **c** Expression of Foxp3, IL-10, and TGF-β after GMSC local treatment in the challenged ears. *n* = 3 independent animals/group. **p < 0.01. *GMSC* gingiva-derived mesenchymal stromal cell, *CHS* contact hypersensitivity

Moreover, the Th1/Th2 balance in the CHS mice was disrupted, changing to a Th1 dominant state, as indicated by the increased IFN-γ/IL-4 ratio. After GMSC infusion, the Th1 state was maintained, but with a significantly decreased IFN-γ/IL-4 ratio (Fig. 7a). Western blot showed a significant decrease in the expression of iNOS in tissue lysates of treated ears when compared with those of untreated CHS (Fig. 7b). TGF-β induces differentiation of naïve T cells into active CD4^+^ Tregs, which express the specific transcription factor Foxp3 and secrete IL-10. To explore the effects of GMSCs on Tregs, the expression of Foxp3, IL-10, and TGF-β in the challenged ears after local GMSC treatment was detected by western blot. This showed significantly increased expression of Foxp3, IL-10, and TGF-β (Fig. 7c).

**Fig. 8:**
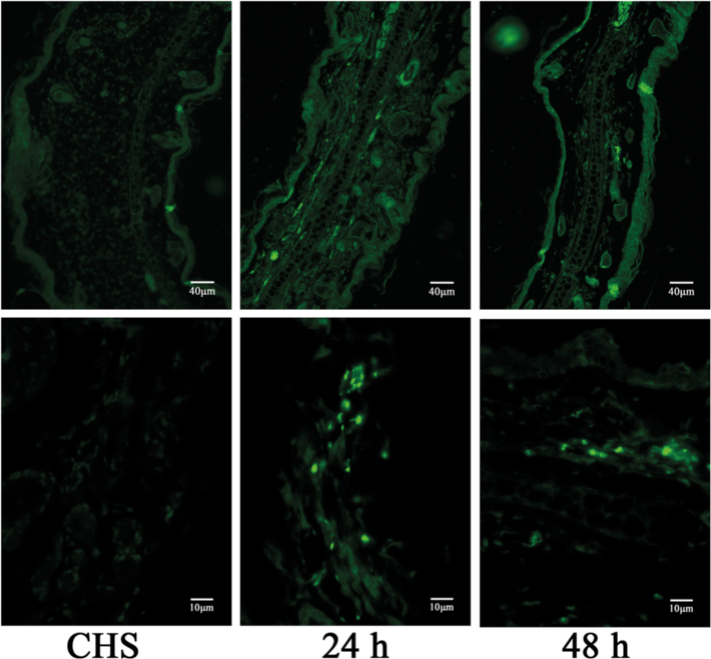
Migration of locally infused GMSCs. Immunofluorescence studies performed using antibodies specific for human leukocyte antigen. *n* = 3 independent animals. *CHS* contact hypersensitivity

We stained HLA in sensitized ears at 24 and 48 h after the local or intravenous injection of GMSCs. Systemically infused GMSCs could not be detected in cross-sections of locally sensitized ears by immunofluorescence staining for HLA. However, subcutaneously applied GMSCs were detected at 24 and 48 h post injection, as determined by HLA expression (Fig. 8).

### GMSCs attenuate CHS via PGE_2_–EP_3_ signaling

**Fig. 9:**
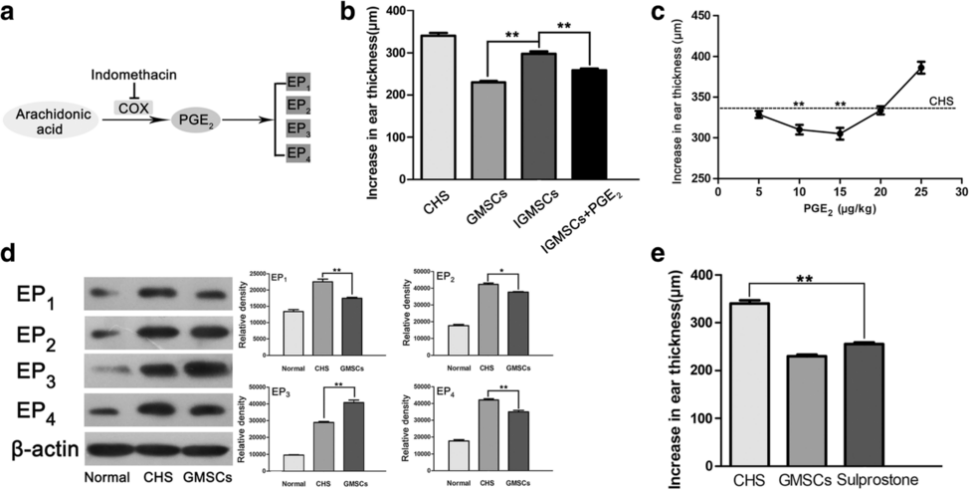
GMSCs attenuate CHS via PGE_2_–EP_3_ signaling. **a** PGE_2_–EP_3_ pathway. **b** IGMSCs significantly reversed the inhibitory effect of GMSCs on CHS appearance, and the application of PGE_2_ and IGMSCs significantly suppressed CHS appearance compared with IGMSCs alone. *n* = 5 independent animals. **c** Subcutaneous administration of PGE_2_ (5–20 μg/kg) led to a dose-dependent suppression of CHS appearance; the suppression of CHS appearance was statistically significant following application of 10 or 15 μg/kg PGE_2_. *n* = 5 independent animals. **d** Western blot showed increased production of EP_3_ and reduced production of EP_1_, EP_2_, and EP_4_ in GMSC-treated local inflammatory tissue. *n* = 3 independent animals/group. **e** Subcutaneous application of sulprotone led to a significant suppression of CHS. *n* = 5 independent animals. *p < 0.05; **p < 0.01. *GMSC* gingiva-derived mesenchymal stromal cell, *IGMSC* indomethacin-pretreated GMSC, *CHS* contact hypersensitivity, *PGE*_*2*_ prostaglandin E_2_

To explore the potential mechanism of the immunomodulatory functions of GMSCs, we studied their relationship with the classical inflammatory pathway via PGE_2_–EP_3_ signaling. IGMSCs significantly, but not completely, reversed the inhibitory effect of GMSCs on CHS appearance (Fig. 9b). Subcutaneous application of dmPGE_2_ (5–20 μg/kg) to CHS mice 1 h after challenge led to a dose-dependent suppression of CHS. The maximum effect was detected at a dose of 15 μg/kg, and the suppression of CHS appearance was statistically significant following application of 10 or 15 μg/kg PGE_2_ (Fig. 9c). In addition, the application of PGE_2_ (15 μg/kg) and IGMSCs incompletely reversed the inadequate CHS suppression induced by IGMSCs alone (Fig. 9b).

Western blot showed increased production of EP_3_ and reduced production of EP_1_, EP_2_, and EP_4_ in local GMSC-treated inflammatory tissue (Fig. 9d). To determine which PGE_2_ receptor mediated the GMSC-mediated immunosuppression of CHS, sulprostone, an agonist of EP_3_, was subcutaneously applied 1 h after challenge and resulted in significant suppression of CHS (Fig. 9e). In addition, the simultaneous application of sulprostone and IGMSCs significantly suppressed CHS appearance, similar to the simultaneous application of PGE_2_ and IGMSCs (see Additional file 4). Taken together, these findings indicated that EP_3_ was associated with the immunomodulatory functions of GMSCs.

## Discussion

The gingiva serves as a mucosal barrier to protect the oral cavity side of the maxilla and mandible; however, it also has some unique features. For example, wound healing within the gingiva and oral mucosa is characterized by markedly reduced inflammation, rapid re-epithelialization, and fetal-like scarless healing, in stark contrast to the common scar formation present in skin [7]. The gingiva might thus also have an immune profile associated more with mucous membranes than with bone marrow or adipose tissue, and therefore GMSCs may exhibit differences in immunological response compared with bone marrow or adipose mesenchymal stromal cells. GMSCs as a novel type of MSCs express lower MSC-related markers than BMSCs and ASCs (flow cytometry of ASCs see Additional file 5), which is consistent with previous reports [10, 11, 33]. MSCs possess potent immunomodulatory functions both in vitro and in vivo [34]. Recent research suggests that GMSCs exert similar immunosuppressive effects in vitro and in vivo [8, 10, 11, 35]. Here, we found that application of GMSCs, ASCs, and BMSCs suppressed the symptoms of CHS more effectively compared with the application of 0.025 % triamcinolone acetonide acetate, and that GMSC administration exhibited the greatest treatment efficacy. Topical corticosteroids, as a standard therapy for CHS, did not achieve the expected results. Because the therapeutic effect of topical corticosteroids is related to the type, concentration, and dosage of drug, the method and the probably insufficient dosage we used may contribute to this insufficient effect. Topical corticosteroid treatment is therefore an inadequate positive control for CHS treatment.

The enhanced treatment effect in CHS and the lower expression of MSC-related markers of GMSCs might be associated with their unique developmental source. A previous study [33] showed that, apart from 10 % of GMSCs from the mesoderm, 90 % of GMSCs are derived from cranial neural crest cells, and show an elevated capacity to induce activated T-cell apoptosis in vitro and upregulation of the expression FAS ligand (FASL), a transmembrane protein that plays an important role in MSC-based immunomodulation [33]. In addition, in mice with dextran sulfate sodium (DSS)-induced experimental colitis, GMSCs derived from cranial neural crest cells resulted in better therapeutic effects with regards to body weight loss, diarrhea, bleeding, histological recovery of the epithelial structure, and the numbers of inflammatory cells, Tregs, and Th17 cells compared with those from mesoderm. This may result in the difference between GMSCs and other types of MSCs. Several comparative studies on the immunomodulatory functions of ASCs and BMSCs have been published [13, 36, 37]; however, the present study is the first to compare the immunomodulatory efficacy of GMSCs, ASCs, and BMSCs in vivo. Our findings suggest that GMSCs are an effective immunotherapeutic tool due to their more powerful immunomodulatory effect and accessibility, which might have important implications in clinical practice. However, there are few related reports in the literature, and the underlying mechanism of the powerful immunomodulatory function of GMSCs remains largely unknown; further studies are therefore needed.

Regarding the administration time of GMSCs in the treatment of CHS, Su et al. [17] showed that prophylactic administration of GMSCs (intervention before sensitization) exhibited greater efficacy. However, while prophylactic administration of GMSCs might benefit some patients with chronic ACD, therapeutic administration of GMSCs is needed more urgently and may have more significant clinical importance. In contrast, although MSCs have been intravenously applied to treat a variety of immune-related and inflammation-related diseases in many clinical trials, how MSCs target specific tissues is largely unknown. This may be why clinical dosing currently includes high numbers of cells [38]. Despite the systemic immunoregulatory function of intravenously infused MSCs, the cells migrate through the circulatory system and finally home in on target sites. The risk of being taken out of circulation and the long distance to target sites may delay or prevent their local function at such sites [39]. Greater consideration of direct MSC administration is therefore needed to target sites where MSCs are located immediately, avoiding passage through the vascular system and the release of bioactive factors acting on surrounding tissue. However, for most target sites, it is usually difficult to apply MSCs directly. An exception to this is the skin, which is superficial and has a large surface area for direct MSC administration. In research comparing the efficacy between systemic and local MSC therapy for diabetic wound healing, local MSC therapy achieved a better effect [25]. Therefore, to enhance the treatment effect of therapeutic administration of GMSCs to CHS, we compared the effects of local injection with those of intravenous injection. We found that local administration exerted a positive effect when intervention was performed later in the course of the disease. Local infusion is therefore the superior option for therapeutic administration of GMSCs. Locally injected GMSCs can function in a more effective manner. To date this is the first exploratory work to focus on the function and effect of locally applied GMSCs, and it might have profound implications in clinical practice. Further studies should address the practical feasibility of local application of MSC-based therapy for the prevention and treatment of allergic diseases, including allergic rhinitis [15] and asthma [40], because even intravenous administration of MSCs before sensitization also exerts significant ameliorative effects in mouse models.

CHS is categorized as a type IV or a delayed type hypersensitivity reaction, which involves a wide range of innate and adaptive immune cells and inflammatory cytokines [41, 42]. In our study, GMSC administration resulted in a significant reduction in inflammatory cell infiltration and the levels of various proinflammatory cytokines in local allergic areas, which suggest that GMSCs may target multiple types of innate and adaptive immune cells. At the same time, GMSCs inhibited inflammation by promoting the induction and functions of Tregs, as evidenced by increased production of Foxp3, IL-10, and TGF-β. These results are consistent with reports that described the role of Tregs in preventing the development of allergic reactions and limiting the magnitude of the inflammatory process [43, 44]. However, the difference in the circulating and local levels of inflammatory cytokines may be related to the difference in test time and the reduced absorption of GMSCs into the circulation after local injection. Moreover, GMSCs promoted the reconstitution of the disrupted Th1/Th2 balance, as determined by the decreased IFN-γ/IL-4 ratio. This result provides evidence for the capacity of GMSCs to induce immunologic reactions to restore homeostasis. The reduction of inflammatory cytokines and the modified Th1/Th2 balance in murine serum provide evidence that locally applied MSCs have a systemic immunoregulatory function, which conflicts with research in which intramuscular MSC injection was applied to treat rats with diabetic polyneuropathy [22]. This previous study found that MSCs remained at the transplant sites without having any systemic effects. Therefore, further studies are warranted to dissect the detailed mechanisms by which locally and intravenously applied GMSCs affect the complex interactions among these immune cells during the course of CHS.

iNOS is a messenger molecule with diverse functions throughout the body. It has nitrosylase activity and mediates cysteine S-nitrosylation of cytoplasmic target proteins such as COX2 [45]. In fact, it has been revealed that MSC-mediated immunosuppression varies among different mammalian species; IDO mediates immunosuppression by human MSCs, while iNOS plays a similar role in mouse MSCs [46]. Our study indicated that the immunomodulatory functions of GMSCs may be associated with the decreased expression of iNOS in mice with CHS. However, the exact mechanism by which MSCs act on iNOS requires further research.

We did not detect systemically infused GMSCs in locally sensitized ears at 24 and 48 h after challenge. Systemically infused MSCs risk being taken out of circulation, on either a temporary or a permanent basis, in organs such as the lungs, spleen, and liver [38]. Upon reaching target sites, MSCs must exit the vasculature to enter the connective tissue stromal region where their principal functions occur [39]. There is ample evidence of systemically infused MSC homing in on local organs, especially the bone marrow, lungs, spleen, and liver [47]. With regards to the skin, systemically infused GMSCs were detected in healing wounds 7 days after cell injection in mice with cutaneous wounds [8]. A report has also described that GMSCs were detected in mucosa 5 days after intravenous injection in mice with chemotherapy-induced oral mucositis [10]. Thus, we infer that, in our study, few homing cells may have been associated with the examined time period. In addition, the heterogeneous source of cells may accelerate MSC deletion. Therefore, we speculate that intravenously applied GMSCs play an anti-inflammatory role through systemic immunomodulation. Subcutaneously applied GMSCs were detected at 24 and 48 h, which provides evidence that locally applied MSCs can migrate to surrounding tissues and function there in a more efficient manner. The difference in the number of GMSCs between 24 and 48 h may be related to the examined position of the ears.

Despite heterogeneous GMSCs being used in this study, no obvious symptoms of xenorejection were observed in the treated mice. In fact, some studies have reported the administration of human MSCs in mouse disease models without immunosuppression. For example, Stoff et al. [48] injected concentrated human MSCs into sites adjacent to incisional wounds made in the skin of rabbits, which resulted in enhanced wound healing. The rabbits used in that study were fully immunocompetent, and no immunosuppressive drugs were given. Moreover, human GMSCs were intravenously injected to treat CHS [17] and experimental colitis [7] in mice (C57BL/6 mice) without the application of immunosuppressive drugs, which produced good results. In addition, research reported that MSCs might increase tolerance for the engraftment of skin equivalents constructed from allogeneic cells and help to promote vascular ingrowth into the graft [49–51]. Notably, no evidence has been reported for the rejection of injected xenogeneic human MSCs [39]. Possible reasons for this include the low immunogenicity and immunomodulatory functions of MSCs.

Generally, PGE_2_ is considered an inflammatory mediator, but it plays a complex role in the development of allergic reactions. Our study reveals that the reduced level of GMSC-derived PGE_2_ induced by indomethacin (an inhibitor of cyclooxygenase) reversed the majority of the inhibition of CHS by GMSCs. These results indicate that PGE_2_ is necessary for GMSC-induced immunomodulation and GMSCs may suppress inflammatory reactions by autocrine PGE_2_. The combined application of PGE_2_ and IGMSCs partially reversed the effect of indomethacin pretreatment. The dose-dependent suppression of CHS by low-dose PGE_2_ administration indicates that endogenous PGE_2_ acts in situ in the skin to modulate the extent of CHS-induced inflammation. All of these findings indicate that PGE_2_ is one of the most important factors mediating the immunomodulatory functions of GMSCs. The therapeutic effects of PGE_2_ on skin allergic inflammation are dependent on its interactions with the various receptor subtypes. In the present study, GMSC administration inhibited the expression of EP_1_, EP_2_, and EP_4_ and promoted that of EP_3._ Sulprostone is an agonist of EP_3_ and EP_1_, with considerably greater affinity for EP_3_ than for EP_1_ (Ki = 0.6 and Ki = 21, respectively) [52], and sulprostone application led to significant suppression of CHS, similar to GMSC application. These findings suggest that stimulation of EP_3_ with an exogenously added agonist can control allergic inflammation in the skin. PGE_2_–EP_3_ signaling plays an important role in the immunomodulatory functions of GMSCs in murine CHS. However, the molecular mechanisms underlying the immunomodulatory effect of PGE_2_–EP_3_ signaling require further research.

Like COX, arachidonic acid is also metabolized by lipoxygenase. As mentioned, NSAIDs adversely affect the diversion from arachidonate metabolism to the lipoxygenase pathway, leading to an increase in leukotriene B4. However, our study suggests another explanation for the adverse effects of NSAIDs in inflammatory skin diseases: NSAIDs weaken the anti-inflammatory effects of PGE_2_–EP_3_ signaling.

## Conclusions

In summary, this study showed, for the first time, that PGE_2_–EP_3_ signaling plays an important role in the immunomodulatory functions of GMSCs in murine CHS. In addition, local infusion, which led to more marked attenuation of CHS during the late phase of disease, is the superior option for therapeutic administration of GMSCs. Furthermore, compared with other types of MSCs, more powerful immunomodulatory functions and accessibility render GMSCs a preferable novel immunotherapeutic.

## Abbreviations

ACD, allergic contact dermatitis; ASC, adipose-derived stem cell; BMSC, bone marrow-derived mesenchymal stem cell; CHS, contact hypersensitivity; GMSC, gingiva-derived mesenchymal stromal cell; IGMSC, indomethacin-pretreated mesenchymal stem cell; MSC, mesenchymal stem cell; NSAID, nonsteroidal anti-inflammatory drug; PGE_2_, prostaglandin E_2_; Treg, regulatory T cell

## Additional files

Additional file 1 The isolaton and culture of all types of cells in the experiment. (PDF 188 kb) Additional file 2 The location of local injection. (TIF 5330 kb) Additional file 3 Details of the therapeutic effects of various MSC administration methods. (PDF 327 kb) Additional file 4 The therapeutic effects of the simultaneous application of sulprotone and IGMSCs. (PDF 83 kb) Additional file 5 Flow cytometry of ASCs. (PDF 1297 kb)
