# Supplementary material for: Therapeutic effects of human gingiva-derived mesenchymal stromal cells on murine contact hypersensitivity via prostaglandin E2–EP3 signaling
Source: Stem Cell Res Ther. 2016 Aug 2;7:103. doi: 10.1186/s13287-016-0361-9 (PMC4969691; doi:10.1186/s13287-016-0361-9)
Supplement: Additional file 1 — The isolaton and culture of all types of cells in the experiment. (PDF 188 kb) [file 13287_2016_361_MOESM1_ESM.pdf]

Cell isolation and culture

The clinically healthy gingiva were collected from routine dental procedures as remnants or discarded tissues, alveolar bone marrow was aspirated from patients undergoing routine dental implant placement at Peking University School and Hospital of Stomatology, and human adipose tissues were obtained from healthy patients who were under liposuction surgery for esthetic reason at Peking University Third Hospital, following approved guidelines set by the Health Science Center, Peking University (PKUSSIRB-201311108).

1. GMSCs isolation and culture

Gingiva were collected from routine dental procedures as remnants or discarded tissues at Peking University School and Hospital of Stomatology. Volunteers aged 18–30 years had no history of periodontal disease and relatively healthy periodontium. Gingival tissues were treated aseptically and incubated overnight at 4 ℃ with dispase (2 mg/ml; Sigma -Aldrich) to separate the epithelial and lower spinous layers. The tissues were minced into 1- to 3-mm^2 fragments and digested at 37 ℃ for 2 h in sterile PBS containing 4 mg/ml collagenase IV (Worthington Biochemical). The dissociated cell suspension was filtered through a 70-μm cell strainer (Falcon, BD Biosciences, San Jose, CA, USA), plated on untreated 10-cm Petri dishes (Costar, Corning Life Sciences, Tewksbury, MA, USA) with complete α-modified Eagle's medium (a-MEM, GIBCO/BRL, Grand Island, NY, USA)) containing 10% fetal bovine serum (FBS; Hyclone Thermo Scientific,Logan, UT, USA), 100 U/ml penicillin/100 μg/ml streptomycin (Sigma, St. Louis, USA), then cultured at 37 ℃ in a humidified tissue culture incubator with 5% CO_2 and 95% O_2.

2. ASCs isolation and culture

Human adipose tissues were obtained from five healthy patients who were under liposuction surgery for esthetic reason at Peking University School and Hospital of Stomatology. The liposuction tissue digested with 0.075% type I collagenase for 60 min at 37 ℃ with intermittent shaking. The floating adipocytes were separated from the stromal cells by centrifugal force (300*g*) for 10 min and the cell pellet was obtained. Then the pellet was resuspended in 160 mM NH_4Cl and incubated at room temperature (RT) for 10 min to lyse contaminating red blood cells. The stromal cells were collected by centrifugation, filtered through a 70-μm cell strainer and were cultured in fresh Dulbecco's modified Eagle's medium (DMEM Gibco, USA) containing 10% FBS, 100 U/mL penicillin G and 100 μg/mL streptomycin at 37 ℃ in an incubator with 5% CO_2 and 95% O_2.

3. BMSCs isolation and culture

Alveolar bone marrow was aspirated from patients undergoing routine dental implant placement. The bone marrow was mixed with 10 ml PBS, and then centrifuged at 1000 rpm for 15 min. Nucleated cells were re-suspended in DMEM; containing 10%

FBS. Cell suspension was incubated at 37 ℃ in a humidified atmosphere of 95% air and 5% CO2.

4. Donors information

**Donors information**	**BMSC**	**ASC**	**GMSC**
**Quantity**	1	1	3
**Gender**	male	male	male
**Age**	26	28	20, 24, 27
**Health status**	Healthy, no history of systemic disease	Healthy, no history of systemic disease BMI=21.34	Healthy, no history of systemic disease and periodontal disease
**Position where the materials were drawn**	Alveolar bone marrow aspirated from dental implant placement of the mandibular first molar	Adipose tissues from liposuction surgery of abdomen for esthetic reasons	Gingiva collected from the extraction of the third molars as remnants tissues

5. Skin-derived fibroblasts isolation and culture

Facial skin were obtained from a 26 year-old man undergoing orthognathic surgery. The connective tissues and adipose tissues were removed from the skin tissues. Then the tissues were minced into 1- to 3-mm2 fragments and digested at 4 ℃ in in fresh Dulbecco's modified Eagle's medium (DMEM Gibco, USA) with 200U/ml collagenase and 300U/ml hyaluronidase dispase. The tissues were digested at 37 ℃ for 20 min in sterile PBS containing 0.25% trypsogen and 0.02%EDTA. Cells were collected by centrifugation, filtered through a 70-μm cell strainer and were cultured in DMEM containing 15% FBS, 100 U/mL penicillin G and 100 μg/mL streptomycin at 37 °C in an incubator with 5% CO2 and 95% O2.

Antibodies and Reagents

Triamcinolone acetonide acetate Cream (0.025%, Qiangsheng, Beijing, China) Indomethacin, oxazolone (Sigma, St. Louis, MO, http://www.sigmaaldrich.com) Sulprotone (Cayman Chemical, Ann Arbor, Michigan https://www.caymanchem.com) All antibodies used in our experiment were purchased from Abcom (http://www.abcam.com/),
