# Supplementary figures and images for: Therapeutic effects of human gingiva-derived mesenchymal stromal cells on murine contact hypersensitivity via prostaglandin E2–EP3 signaling
Source: Stem Cell Res Ther. 2016 Aug 2;7:103. doi: 10.1186/s13287-016-0361-9 (PMC4969691; doi:10.1186/s13287-016-0361-9)

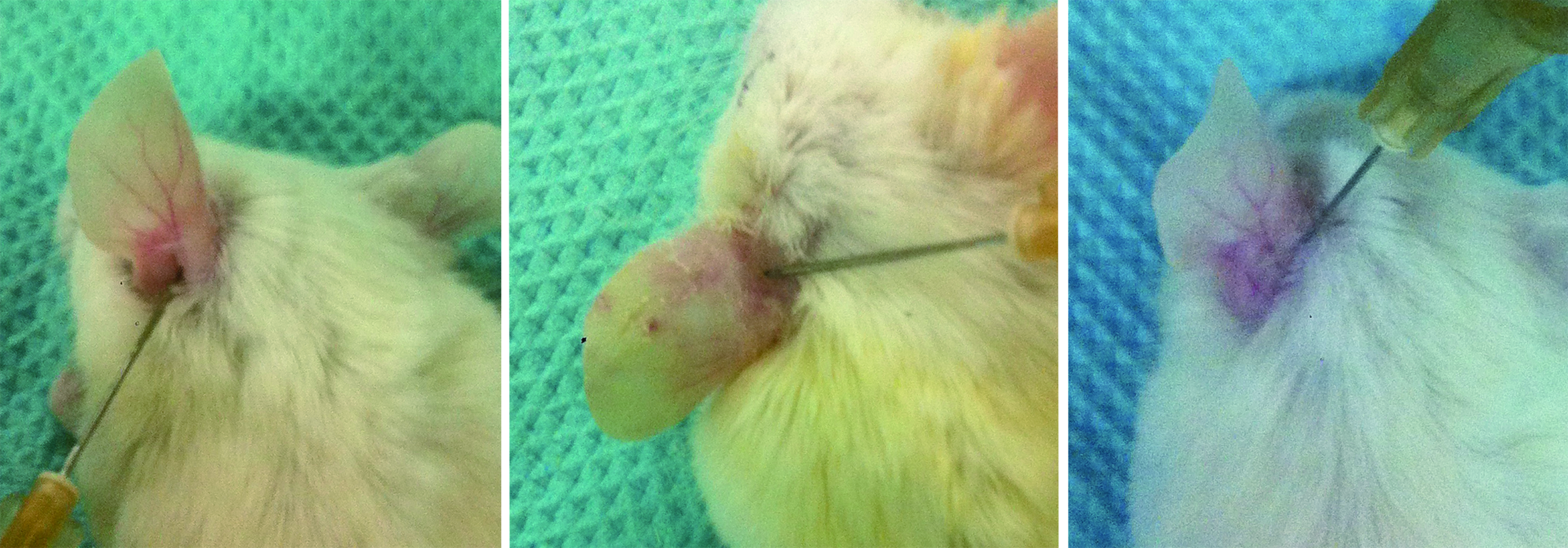

Supplement: Additional file 2 — The location of local injection. (TIF 5330 kb) [file 13287_2016_361_MOESM2_ESM.tif]

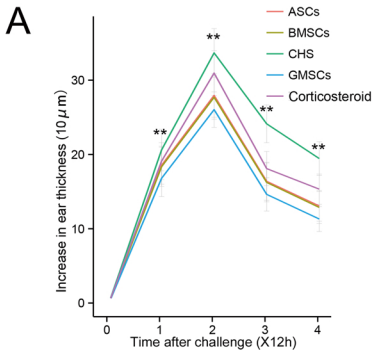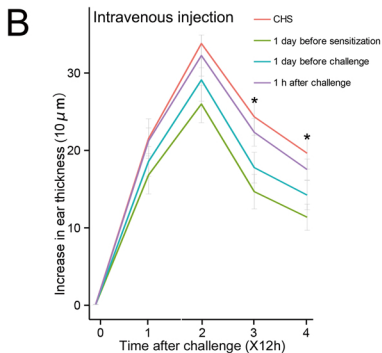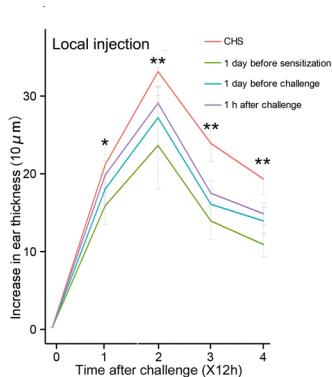

Supplement: Additional file 3 — Details of the therapeutic effects of various MSC administration methods. (PDF 327 kb) [file 13287_2016_361_MOESM3_ESM.pdf]

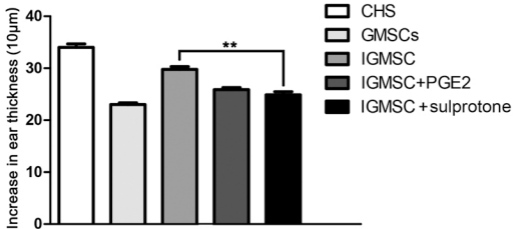

Supplement: Additional file 4 — The therapeutic effects of the simultaneous application of sulprotone and IGMSCs. (PDF 83 kb) [file 13287_2016_361_MOESM4_ESM.pdf]

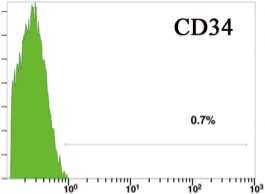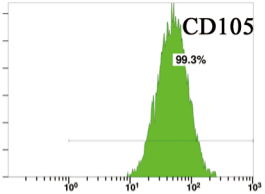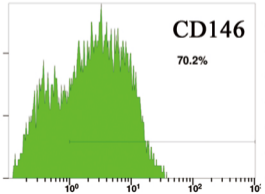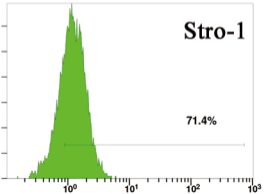

Supplement: Additional file 5 — Flow cytometry of ASCs. (PDF 1297 kb) [file 13287_2016_361_MOESM5_ESM.pdf]
